# Benzyl *N*-{(1*S*)-2-hy­droxy-1-[*N*′-(2-nitro­benzyl­idene)hydrazinylcarbon­yl]eth­yl}carbamate

**DOI:** 10.1107/S1600536810027273

**Published:** 2010-07-14

**Authors:** Marcus V. N. de Souza, Alessandra C. Pinheiro, Edward R. T. Tiekink, Solange M. S. V. Wardell, James L. Wardell

**Affiliations:** a Fundação Oswaldo Cruz, Instituto de Tecnologia em Fármacos – Farmanguinhos, R. Sizenando Nabuco, 100, Manguinhos, 21041-250 Rio de Janeiro, RJ, Brazil; b Department of Chemistry, University of Malaya, 50603 Kuala Lumpur, Malaysia; c CHEMSOL, 1 Harcourt Road, Aberdeen AB15 5NY, Scotland; d Centro de Desenvolvimento Tecnológico em Saúde (CDTS), Fundação Oswaldo Cruz (FIOCRUZ), Casa Amarela, Campus de Manguinhos, Av. Brasil 4365, 21040-900 Rio de Janeiro, RJ, Brazil

## Abstract

The carbamate and hydrazone groups in the title compound, C_18_H_18_N_4_O_6_, are approximately orthogonal [dihedral angle = 83.3 (4)°], and the carbonyl groups are effectively *anti* [O=C⋯C=O torsion angle = −116.2 (7)°]. The conformation about the imine bond [1.295 (11) Å] is *E*. The crystal packing is dominated by O—H⋯O and N—H⋯O hydrogen bonding, which leads to two-dimensional arrays in the *ab* plane.

## Related literature

For background to the anti-tumour potential of l-serine derivatives, see: Jiao *et al.* (2009 ▶); Yakura *et al.* (2007 ▶); Takahashi *et al.* (1988 ▶); Sin *et al.* (1998 ▶). For background of the anti-tumour potential of *N*-acyl­hydrazone l-serine derivatives, see: Rollas & Küçükgüzel (2007 ▶); Terzioğlu & Gürsoy (2003 ▶).
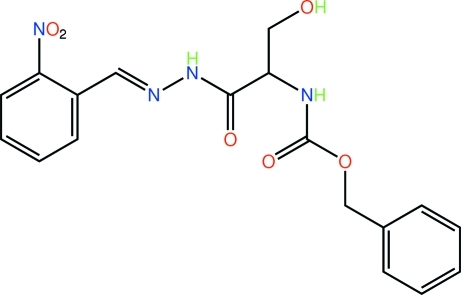


## Experimental

### 

#### Crystal data

C_18_H_18_N_4_O_6_
*M* *_r_* = 386.36
Triclinic, 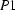

*a* = 4.6675 (7) Å
*b* = 5.7001 (7) Å
*c* = 16.645 (3) Å
α = 90.457 (9)°
β = 92.087 (7)°
γ = 97.319 (9)°
*V* = 438.90 (11) Å^3^
*Z* = 1
Mo *K*α radiation
μ = 0.11 mm^−1^
*T* = 120 K
0.20 × 0.07 × 0.01 mm

#### Data collection

Nonius KappaCCD area-detector diffractometer
Absorption correction: multi-scan (*SADABS*; Sheldrick, 2007 ▶) *T*_min_ = 0.624, *T*_max_ = 1.000
7083 measured reflections
1791 independent reflections
1243 reflections with *I* > 2σ(*I*)
*R*_int_ = 0.093

#### Refinement

*R*[*F* ^2^ > 2σ(*F* ^2^)] = 0.089
*wR*(*F* ^2^) = 0.190
*S* = 1.14
1791 reflections
262 parameters
6 restraints
H atoms treated by a mixture of independent and constrained refinement
Δρ_max_ = 0.39 e Å^−3^
Δρ_min_ = −0.32 e Å^−3^

### 

Data collection: *COLLECT* (Hooft, 1998 ▶); cell refinement: *DENZO* (Otwinowski & Minor, 1997 ▶) and *COLLECT*; data reduction: *DENZO* and *COLLECT*; program(s) used to solve structure: *SHELXS97* (Sheldrick, 2008 ▶); program(s) used to refine structure: *SHELXL97* (Sheldrick, 2008 ▶); molecular graphics: *ORTEP-3* (Farrugia, 1997 ▶) and *DIAMOND* (Brandenburg, 2006 ▶); software used to prepare material for publication: *publCIF* (Westrip, 2010 ▶).

## Supplementary Material

Crystal structure: contains datablocks global, I. DOI: 10.1107/S1600536810027273/zs2049sup1.cif

Structure factors: contains datablocks I. DOI: 10.1107/S1600536810027273/zs2049Isup2.hkl

Additional supplementary materials:  crystallographic information; 3D view; checkCIF report

## Figures and Tables

**Table 1:** Hydrogen-bond geometry (Å, °)

*D*—H⋯*A*	*D*—H	H⋯*A*	*D*⋯*A*	*D*—H⋯*A*
O3—H3o⋯O2^i^	0.84 (8)	1.97 (9)	2.712 (9)	147 (8)
N1—H1n⋯O3^ii^	0.88 (7)	2.12 (7)	2.974 (10)	167 (8)
N2—H2n⋯O4^iii^	0.88 (4)	1.95 (5)	2.775 (10)	155 (9)

Symmetry codes: (i) 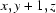; (ii) 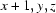; (iii) 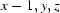.

**Fig. 1.**
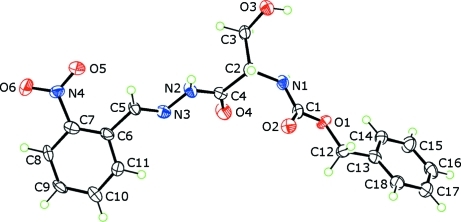
The molecular structure of (I) showing the atom-labelling scheme and displacement ellipsoids at the 50% probability level.

**Fig. 2.**
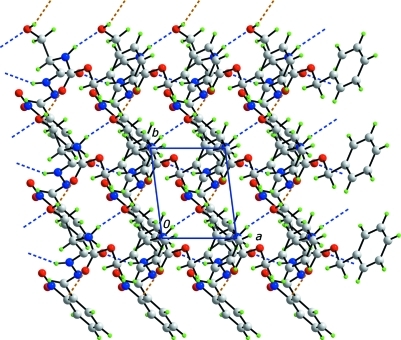
A view of the two-dimensional supramolecular array in the ab plane in (I) with the O–H···O and N–H···O hydrogen bonding shown as orange and blue dashed lines, respectively.

**Fig. 3.**
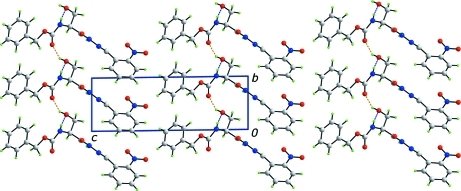
A view in projection down the a axis of (I) showing the stacking of the two-dimensional arrays along the c axis.

## supplementary crystallographic information

### Comment

Several *L*-serine derivatives have been found to have potential in anti-cancer therapy, for example, conagenin, a naturally occurring serine derivative, was shown to improve the anti-tumour efficacy of adriamycin and mitomycin C against murine leukemias (Jiao *et al.*, 2009; Yakura *et al.*, 2007). Other *L*-serine derivatives reported as potential new anti-tumour agents include the antibiotic thrazarine, which sensitizes tumour cells to macrophage-mediated cytolysis (Takahashi *et al.*, 1988), and eponemycin, an immunomodulator, which plays a crucial role in tumour progression and metastases by supplying essential nutrients to B16 melanoma cells (Sin *et al.*, 1998).

Following on from such reports, we have synthesized some *N*-acylhydrazone *L*-serine derivatives from *L*-serine to screen for anti-tumour activity. The choice of *N*-acylhydrazonyl derivatives was suggested by publications indicating that compounds with such groups can aid anti-tumour activities (Rollas & Küçükgüzel, 2007; Terzioğlu & Gürsoy, 2003). We now report the structure of the title compound (I). While the solid, isolated by recrystallization from methanol, was purely in the *E*-form, NMR spectra in DMSO-d6 solution indicated that both *E* and *Z* forms are produced.

Significant twists are evident in the molecular structure of (I) (Fig. 1). The twisting is most pronounced about the central methine link with the dihedral angle formed between the least-squares planes through the carbamate group (N1,C1,O1,O2; r.m.s. = 0.0028 Å) and the hydrazone group (N2,N2,C4,O4; r.m.s. = 0.0202 Å) being 83.3 (4)°. The dihedral angle O2–C2···C4–O4 is -116.2 (7)° indicating an *anti* disposition for the carbonyl-O2 and O4 atoms. While the benzyl-benzene ring is approximately co-planar with the carbamate group [the O1–C1–C12–C13 torsion angle is -171.3 (8)°], the benzene ring adjacent to the hydrazone residue is not [N3–C5–C6–C7 = -137.9 (9) °]; the dihedral angle formed between the terminal benzene rings is 67.8 (4) °. The conformation about the imine C5═N3 bond [1.295 (11) Å] is *E*.

The crystal packing is dominated by O–H···O and N–H···O hydrogen bonding (Table 1). The hydroxyl group hydrogen bonds with the carbonyl-O2 to form a chain along the *b* axis. Each N–H hydrogen-bonds to an O atom, N1–H to the hydroxy-O3 atom to form a chain along the *a* axis, and N3–H to a carbonyl-O4 atom to form an amide-type tape along the *a* axis. The net result of the hydrogen bonding is the formation of two-dimensional arrays in the *ab* plane (Fig. 2), that stack along the *c* axis (Fig. 3).

### Experimental

An ethanolic solution of 2-nitrobenzaldehyde (1.05 mmol) and PhCH_2_O(CO)NHCH(CH_2_OH)CONHNH_2_, prepared from *L*-serine and hydrazine hydrate, (1.0 mmol) was refluxed for 4 h. After rotary evaporation, the residue was washed with cold ethanol (3 *x* 10 ml), and recrystallized from methanol. The crystals used in the structure determination were grown from methanol solution, m.p. 428–429 K.

^1^H NMR (500 MHz, DMSO-d6) δ (p.p.m.): 11.88 and 11.71 (1*H*, s, NHN, (E/*Z*)-diastereomer), 8.66 and 8.39 (1*H*, s, N═CH, (E/*Z*)-diastereomer), 8.07 (2*H*, m, H3 and H6), 7.80 (1*H*, m, H5), 7.67 (1*H*, m, H4), 7.44 (d, J = 7.8) and 7.34 (*m*), (1*H*, NHCH, (E/*Z*)-diastereomer), 7.38–7.30 (5*H*, m, Ph), 5.05 and 5.04 (2*H*, s, CH_2_Ph, (E/*Z*)-diastereomer), 5.03 (*m*) and 4.89 (t, J = 5.9), (1*H*, OH, (E/*Z*)-diastereomer), 5.03 and 4.15 (1*H*, m, CH, (E/*Z*)-diastereomer), 3.80–3.60 (2*H*, m, CH_2_OH). ^13^C NMR (125 MHz, DMSO-d6) δ (p.p.m.): 171.7, 167.4, 156.0, 148.2, 148.1, 142.4, 138.8, 137.0, 136.9, 133.8, 133.6, 130.6, 130.5, 128.7, 128.4, 128.0, 127.8, 127.7, 124.7, 124.6, 65.6, 65.4, 61.4, 61.1, 56.5, 54.4. IR (cm^-1^; KBr): 3392 (O—H), 1694 (COCH), 1672 (COO), 1555 and 1342 (NO_2_). EM/ESI: [M—H]: 385.3.

### Refinement

The C-bound H atoms were geometrically placed (C–H = 0.95–1.00 Å) and refined as riding with *U*_iso_(H) = 1.2*U*_eq_(C). The O– and N-bound H atoms were located from a difference map and refined with the distance restraints O–H = 0.84±0.01 and N–H = 0.88±0.01 Å, and with *U*_iso_(H) = 1.2*U*_eq_(N) or 1.5*U*_eq_(O). In the absence of significant anomalous scattering effects, 1489 Friedel pairs were averaged in the final refinement. However, the absolute configuration was assigned on the basis of the chiralty of the *L*-serine starting material.

### Crystal data

**Table:**

C_18_H_18_N_4_O_6_	*Z* = 1
*M**_r_* = 386.36	*F*(000) = 202
Triclinic, *P*1	*D*_x_ = 1.462 Mg m^−^^3^
Hall symbol: P 1	Melting point = 428–429 K
*a* = 4.6675 (7) Å	Mo *K*α radiation, λ = 0.71073 Å
*b* = 5.7001 (7) Å	Cell parameters from 18416 reflections
*c* = 16.645 (3) Å	θ = 2.9–27.5°
α = 90.457 (9)°	µ = 0.11 mm^−^^1^
β = 92.087 (7)°	*T* = 120 K
γ = 97.319 (9)°	Plate, colourless
*V* = 438.90 (11) Å^3^	0.20 × 0.07 × 0.01 mm

### Data collection

**Table:**

Nonius KappaCCD area-detector diffractometer	1791 independent reflections
Radiation source: Enraf Nonius FR591 rotating anode	1243 reflections with *I* > 2σ(*I*)
10 cm confocal mirrors	*R*_int_ = 0.093
Detector resolution: 9.091 pixels mm^-1^	θ_max_ = 26.5°, θ_min_ = 3.6°
φ and ω scans	*h* = −5→5
Absorption correction: multi-scan (*SADABS*; Sheldrick, 2007)	*k* = −6→7
*T*_min_ = 0.624, *T*_max_ = 1.000	*l* = −20→20
7083 measured reflections	

### Refinement

**Table:**

Refinement on *F*^2^	Primary atom site location: structure-invariant direct methods
Least-squares matrix: full	Secondary atom site location: difference Fourier map
*R*[*F*^2^ > 2σ(*F*^2^)] = 0.089	Hydrogen site location: inferred from neighbouring sites
*wR*(*F*^2^) = 0.190	H atoms treated by a mixture of independent and constrained refinement
*S* = 1.14	*w* = 1/[σ^2^(*F*_o_^2^) + (0.037*P*)^2^ + 1.2849*P*] where *P* = (*F*_o_^2^ + 2*F*_c_^2^)/3
1791 reflections	(Δ/σ)_max_ < 0.001
262 parameters	Δρ_max_ = 0.39 e Å^−^^3^
6 restraints	Δρ_min_ = −0.32 e Å^−^^3^

### Special details

**Table:**

Geometry. All s.u.'s (except the s.u. in the dihedral angle between two l.s. planes) are estimated using the full covariance matrix. The cell s.u.'s are taken into account individually in the estimation of s.u.'s in distances, angles and torsion angles; correlations between s.u.'s in cell parameters are only used when they are defined by crystal symmetry. An approximate (isotropic) treatment of cell s.u.'s is used for estimating s.u.'s involving l.s. planes.
Refinement. Refinement of *F*^2^ against ALL reflections. The weighted *R*-factor *wR* and goodness of fit *S* are based on *F*^2^, conventional *R*-factors *R* are based on *F*, with *F* set to zero for negative *F*^2^. The threshold expression of *F*^2^ > 2σ(*F*^2^) is used only for calculating *R*-factors(gt) *etc*. and is not relevant to the choice of reflections for refinement. *R*-factors based on *F*^2^ are statistically about twice as large as those based on *F*, and *R*- factors based on ALL data will be even larger.

### Fractional atomic coordinates and isotropic or equivalent isotropic displacement parameters (Å^2^)

**Table:**

	*x*	*y*	*z*	*U*_iso_*/*U*_eq_	
O1	0.2947 (12)	0.8892 (10)	0.3007 (4)	0.0270 (14)	
O2	−0.0905 (13)	0.6386 (10)	0.2534 (4)	0.0294 (15)	
O3	−0.4518 (12)	1.3215 (11)	0.1633 (4)	0.0297 (15)	
H3O	−0.350 (19)	1.374 (18)	0.204 (4)	0.045*	
O4	0.1745 (14)	0.8327 (12)	0.0625 (4)	0.0395 (17)	
O5	−0.6765 (15)	0.6119 (12)	−0.2286 (4)	0.0427 (19)	
O6	−0.6271 (16)	0.4575 (13)	−0.3452 (4)	0.0450 (19)	
N1	0.0112 (16)	1.0119 (13)	0.2030 (5)	0.0267 (17)	
H1N	0.156 (13)	1.122 (12)	0.195 (6)	0.032*	
N2	−0.2884 (15)	0.7320 (14)	0.0165 (5)	0.0285 (18)	
H2N	−0.469 (7)	0.718 (17)	0.031 (5)	0.034*	
N3	−0.1965 (16)	0.5952 (12)	−0.0441 (5)	0.0272 (18)	
N4	−0.5754 (16)	0.4722 (12)	−0.2727 (5)	0.0291 (18)	
C1	0.0588 (17)	0.8301 (16)	0.2530 (5)	0.024 (2)	
C2	−0.2011 (19)	0.9716 (15)	0.1386 (5)	0.025 (2)	
H2	−0.3785	0.8760	0.1585	0.030*	
C3	−0.278 (2)	1.2117 (16)	0.1085 (5)	0.029 (2)	
H3A	−0.0978	1.3188	0.1004	0.035*	
H3B	−0.3842	1.1877	0.0559	0.035*	
C4	−0.0811 (19)	0.8361 (14)	0.0693 (5)	0.0221 (19)	
C5	−0.398 (2)	0.4987 (15)	−0.0938 (5)	0.026 (2)	
H5	−0.5928	0.5308	−0.0921	0.031*	
C6	−0.3011 (19)	0.3339 (14)	−0.1536 (6)	0.026 (2)	
C7	−0.3789 (19)	0.3147 (14)	−0.2349 (6)	0.026 (2)	
C8	−0.2776 (19)	0.1553 (16)	−0.2870 (6)	0.029 (2)	
H8	−0.3347	0.1492	−0.3424	0.035*	
C9	−0.092 (2)	0.0061 (16)	−0.2560 (6)	0.032 (2)	
H9	−0.0215	−0.1062	−0.2902	0.039*	
C10	−0.006 (2)	0.0190 (16)	−0.1754 (6)	0.031 (2)	
H10	0.1263	−0.0809	−0.1548	0.038*	
C11	−0.1140 (18)	0.1766 (14)	−0.1252 (5)	0.026 (2)	
H11	−0.0600	0.1790	−0.0696	0.031*	
C12	0.347 (2)	0.7141 (16)	0.3589 (6)	0.030 (2)	
H12A	0.4009	0.5724	0.3313	0.036*	
H12B	0.1691	0.6662	0.3884	0.036*	
C13	0.5886 (19)	0.8140 (16)	0.4173 (6)	0.026 (2)	
C14	0.737 (2)	1.0378 (16)	0.4112 (6)	0.030 (2)	
H14	0.6857	1.1380	0.3691	0.035*	
C15	0.958 (2)	1.1178 (18)	0.4651 (6)	0.038 (2)	
H15	1.0609	1.2714	0.4596	0.045*	
C16	1.031 (2)	0.9758 (18)	0.5270 (6)	0.038 (3)	
H16	1.1847	1.0311	0.5642	0.045*	
C17	0.881 (2)	0.7527 (18)	0.5351 (6)	0.037 (3)	
H17	0.9294	0.6546	0.5781	0.045*	
C18	0.659 (2)	0.6729 (16)	0.4798 (6)	0.031 (2)	
H18	0.5543	0.5199	0.4853	0.037*	

### Atomic displacement parameters (Å^2^)

**Table:**

	*U*^11^	*U*^22^	*U*^33^	*U*^12^	*U*^13^	*U*^23^
O1	0.024 (3)	0.030 (3)	0.027 (4)	0.003 (3)	0.002 (3)	0.012 (3)
O2	0.021 (3)	0.029 (3)	0.036 (4)	−0.002 (3)	−0.002 (3)	0.000 (3)
O3	0.023 (4)	0.035 (4)	0.032 (4)	0.003 (3)	0.003 (3)	−0.006 (3)
O4	0.022 (4)	0.049 (4)	0.047 (4)	0.003 (3)	−0.001 (3)	−0.015 (3)
O5	0.048 (5)	0.043 (4)	0.041 (4)	0.024 (4)	−0.002 (4)	−0.006 (3)
O6	0.053 (5)	0.057 (5)	0.027 (4)	0.018 (4)	−0.008 (3)	−0.001 (3)
N1	0.022 (4)	0.032 (5)	0.025 (4)	0.001 (3)	−0.002 (3)	−0.006 (3)
N2	0.016 (4)	0.038 (4)	0.032 (4)	0.003 (3)	0.003 (3)	−0.013 (4)
N3	0.026 (4)	0.024 (4)	0.033 (4)	0.003 (3)	0.003 (4)	0.000 (3)
N4	0.030 (5)	0.022 (4)	0.035 (5)	0.004 (3)	−0.004 (4)	0.005 (3)
C1	0.010 (4)	0.037 (5)	0.023 (5)	0.000 (4)	−0.005 (4)	−0.008 (4)
C2	0.018 (5)	0.028 (5)	0.028 (5)	−0.003 (4)	0.003 (4)	−0.001 (4)
C3	0.039 (6)	0.026 (5)	0.022 (5)	0.003 (4)	−0.005 (4)	−0.004 (4)
C4	0.026 (5)	0.020 (4)	0.021 (5)	0.002 (3)	0.013 (4)	0.005 (3)
C5	0.028 (5)	0.028 (5)	0.022 (5)	0.005 (4)	0.004 (4)	−0.006 (4)
C6	0.026 (5)	0.016 (4)	0.037 (6)	0.000 (4)	−0.004 (4)	−0.007 (4)
C7	0.020 (5)	0.016 (4)	0.041 (6)	−0.004 (3)	0.005 (4)	−0.001 (4)
C8	0.032 (5)	0.034 (5)	0.019 (5)	0.000 (4)	0.000 (4)	−0.011 (4)
C9	0.037 (6)	0.027 (5)	0.036 (6)	0.012 (4)	0.006 (5)	−0.013 (4)
C10	0.032 (5)	0.028 (5)	0.034 (6)	0.000 (4)	0.007 (5)	−0.010 (4)
C11	0.028 (5)	0.023 (5)	0.023 (5)	−0.005 (4)	−0.001 (4)	0.003 (4)
C12	0.031 (5)	0.025 (5)	0.033 (6)	0.005 (4)	−0.005 (4)	0.002 (4)
C13	0.023 (5)	0.030 (5)	0.025 (5)	0.005 (4)	0.011 (4)	0.000 (4)
C14	0.030 (5)	0.030 (5)	0.028 (5)	0.003 (4)	−0.005 (4)	−0.002 (4)
C15	0.039 (6)	0.036 (6)	0.036 (6)	−0.001 (5)	−0.010 (5)	0.000 (5)
C16	0.026 (5)	0.046 (6)	0.040 (6)	0.004 (5)	−0.007 (5)	−0.021 (5)
C17	0.036 (6)	0.043 (6)	0.034 (6)	0.010 (5)	−0.009 (5)	−0.004 (5)
C18	0.034 (6)	0.026 (5)	0.031 (6)	0.000 (4)	−0.001 (4)	0.000 (4)

### Geometric parameters (Å, °)

**Table:**

O1—C1	1.340 (10)	C6—C11	1.402 (12)
O1—C12	1.433 (10)	C7—C8	1.388 (12)
O2—C1	1.218 (11)	C8—C9	1.381 (13)
O3—C3	1.433 (11)	C8—H8	0.9500
O3—H3O	0.84 (8)	C9—C10	1.384 (13)
O4—C4	1.205 (10)	C9—H9	0.9500
O5—N4	1.228 (10)	C10—C11	1.375 (12)
O6—N4	1.223 (10)	C10—H10	0.9500
N1—C1	1.369 (12)	C11—H11	0.9500
N1—C2	1.430 (11)	C12—C13	1.513 (13)
N1—H1N	0.88 (7)	C12—H12A	0.9900
N2—C4	1.358 (11)	C12—H12B	0.9900
N2—N3	1.383 (10)	C13—C14	1.379 (12)
N2—H2N	0.88 (4)	C13—C18	1.376 (13)
N3—C5	1.295 (11)	C14—C15	1.372 (13)
N4—C7	1.490 (11)	C14—H14	0.9500
C2—C4	1.544 (12)	C15—C16	1.376 (15)
C2—C3	1.541 (12)	C15—H15	0.9500
C2—H2	1.0000	C16—C17	1.381 (14)
C3—H3A	0.9900	C16—H16	0.9500
C3—H3B	0.9900	C17—C18	1.391 (13)
C5—C6	1.485 (12)	C17—H17	0.9500
C5—H5	0.9500	C18—H18	0.9500
C6—C7	1.387 (13)		
			
C1—O1—C12	114.3 (7)	C8—C7—N4	115.2 (8)
C3—O3—H3O	110 (7)	C9—C8—C7	118.1 (8)
C1—N1—C2	119.6 (7)	C9—C8—H8	120.9
C1—N1—H1N	118 (6)	C7—C8—H8	120.9
C2—N1—H1N	116 (6)	C10—C9—C8	120.5 (8)
C4—N2—N3	116.5 (7)	C10—C9—H9	119.8
C4—N2—H2N	118 (6)	C8—C9—H9	119.8
N3—N2—H2N	123 (6)	C9—C10—C11	119.9 (9)
C5—N3—N2	115.3 (7)	C9—C10—H10	120.1
O6—N4—O5	123.4 (8)	C11—C10—H10	120.1
O6—N4—C7	119.1 (7)	C10—C11—C6	122.0 (8)
O5—N4—C7	117.5 (7)	C10—C11—H11	119.0
O2—C1—O1	124.3 (8)	C6—C11—H11	119.0
O2—C1—N1	124.7 (8)	O1—C12—C13	109.6 (7)
O1—C1—N1	111.0 (7)	O1—C12—H12A	109.7
N1—C2—C4	109.8 (7)	C13—C12—H12A	109.7
N1—C2—C3	109.1 (7)	O1—C12—H12B	109.7
C4—C2—C3	109.9 (7)	C13—C12—H12B	109.7
N1—C2—H2	109.3	H12A—C12—H12B	108.2
C4—C2—H2	109.3	C14—C13—C18	119.1 (8)
C3—C2—H2	109.3	C14—C13—C12	123.3 (8)
O3—C3—C2	112.7 (7)	C18—C13—C12	117.5 (8)
O3—C3—H3A	109.1	C13—C14—C15	120.9 (9)
C2—C3—H3A	109.1	C13—C14—H14	119.6
O3—C3—H3B	109.1	C15—C14—H14	119.6
C2—C3—H3B	109.1	C16—C15—C14	120.1 (10)
H3A—C3—H3B	107.8	C16—C15—H15	120.0
O4—C4—N2	124.3 (8)	C14—C15—H15	120.0
O4—C4—C2	121.9 (8)	C15—C16—C17	120.0 (9)
N2—C4—C2	113.7 (7)	C15—C16—H16	120.0
N3—C5—C6	114.6 (8)	C17—C16—H16	120.0
N3—C5—H5	122.7	C16—C17—C18	119.4 (10)
C6—C5—H5	122.7	C16—C17—H17	120.3
C7—C6—C11	115.8 (8)	C18—C17—H17	120.3
C7—C6—C5	127.2 (8)	C13—C18—C17	120.5 (9)
C11—C6—C5	117.0 (8)	C13—C18—H18	119.7
C6—C7—C8	123.6 (8)	C17—C18—H18	119.7
C6—C7—N4	121.2 (7)		
			
C4—N2—N3—C5	−179.8 (9)	O6—N4—C7—C6	176.4 (9)
C12—O1—C1—O2	−5.2 (12)	O5—N4—C7—C6	−3.3 (12)
C12—O1—C1—N1	175.7 (7)	O6—N4—C7—C8	−2.2 (11)
C2—N1—C1—O2	−9.2 (13)	O5—N4—C7—C8	178.1 (8)
C2—N1—C1—O1	169.9 (7)	C6—C7—C8—C9	0.7 (13)
C1—N1—C2—C4	−76.5 (9)	N4—C7—C8—C9	179.2 (8)
C1—N1—C2—C3	163.0 (8)	C7—C8—C9—C10	−1.0 (14)
N1—C2—C3—O3	−73.3 (9)	C8—C9—C10—C11	1.9 (14)
C4—C2—C3—O3	166.2 (7)	C9—C10—C11—C6	−2.6 (14)
N3—N2—C4—O4	6.5 (13)	C7—C6—C11—C10	2.2 (12)
N3—N2—C4—C2	−176.0 (7)	C5—C6—C11—C10	−178.9 (8)
N1—C2—C4—O4	−20.2 (11)	C1—O1—C12—C13	−171.3 (8)
C3—C2—C4—O4	99.8 (10)	O1—C12—C13—C14	−2.3 (12)
N1—C2—C4—N2	162.3 (8)	O1—C12—C13—C18	177.0 (8)
C3—C2—C4—N2	−77.7 (9)	C18—C13—C14—C15	2.0 (14)
N2—N3—C5—C6	−174.3 (7)	C12—C13—C14—C15	−178.8 (9)
N3—C5—C6—C7	−137.9 (9)	C13—C14—C15—C16	−1.1 (15)
N3—C5—C6—C11	43.3 (12)	C14—C15—C16—C17	−0.2 (15)
C11—C6—C7—C8	−1.3 (12)	C15—C16—C17—C18	0.6 (15)
C5—C6—C7—C8	179.9 (9)	C14—C13—C18—C17	−1.6 (14)
C11—C6—C7—N4	−179.7 (8)	C12—C13—C18—C17	179.2 (9)
C5—C6—C7—N4	1.5 (13)	C16—C17—C18—C13	0.3 (15)

### Hydrogen-bond geometry (Å, °)

**Table:**

*D*—H···*A*	*D*—H	H···*A*	*D*···*A*	*D*—H···*A*
O3—H3o···O2^i^	0.84 (8)	1.97 (9)	2.712 (9)	147 (8)
N1—H1n···O3^ii^	0.88 (7)	2.12 (7)	2.974 (10)	167 (8)
N2—H2n···O4^iii^	0.88 (4)	1.95 (5)	2.775 (10)	155 (9)

Symmetry codes: (i) *x*, *y*+1, *z*; (ii) *x*+1, *y*, *z*; (iii) *x*−1, *y*, *z*.
